# A matter of balance

**DOI:** 10.7554/eLife.40034

**Published:** 2018-08-21

**Authors:** Aaron D Gitler, John D Fryer

**Affiliations:** 1 Department of GeneticsStanford University School of MedicineStanfordUnited States; 2 Department of NeuroscienceMayo ClinicJacksonvilleUnited States

**Keywords:** Amyotrophic Lateral Sclerosis, Frontotemporal Dementia, mRNA splicing, RNA binding proteins, TD-43, C9orf72, Human

## Abstract

New analyses shift the view that some forms of amyotrophic lateral sclerosis and frontotemporal dementia are due to defects in a single RNA-binding protein.

**Related research article** Conlon EG, Fagegaltier D, Agius P, Davis-Porada J, Gregory J, Hubbard I, Kang K, Kim D, Phatnani H, Shneider NA, Manley JL, New York Genome Center ALS Consortium. 2018. Unexpected similarities between C9ORF72 and sporadic forms of ALS/FTD suggest a common disease mechanism. *eLife* **7**:e37754. doi: 10.7554/eLife.37754

Amyotrophic lateral sclerosis (ALS) is a cruel disease where the death of motor neurons in the brain and spinal cord causes loss of mobility, dexterity, speech, and the ability to swallow. ALS is ultimately fatal, usually within about three years of the onset of symptoms (Taylor et al., 2016). In another neurodegenerative disease, frontotemporal dementia (FTD), neurons from the frontal and temporal lobes of the brain die, leading to a range of cognitive and behavioral symptoms such as personality changes (Ling et al., 2013). The clinical overlap between ALS and FTD has become apparent in recent years: for example, clumps of an RNA-binding protein called TDP-43 build up in almost all cases of ALS and half of FTD cases (Neumann et al., 2006). In 2011, the discovery that a mutation in a gene called *C9orf72* is the most common genetic cause of both ALS and FTD confirmed the connection between the diseases (DeJesus-Hernandez et al., 2011; Renton et al., 2011; ).

*C9orf72* is a peculiar gene – its name literally stands for the 72^nd^ open reading frame on chromosome 9 – and the disease-causing mutation is even stranger. One of the gene's introns normally contains between two and 23 repeats of a six-nucleotide segment (GGGGCC), but this number can rise to hundreds (or even thousands) in the mutant gene. This increase in repeat numbers could cause disease in several ways: the repeated segments could, for example, be transcribed into RNA molecules that soak up RNA-binding proteins and prevent these proteins from carrying out their functions (Gitler and Tsuiji, 2016).

Researchers have identified RNA-binding proteins that might be disrupted in this way. In a previous eLife paper, researchers at Columbia University reported that one of these proteins, called hnRNP H, attaches to the repeat RNA in *C9orf72* and forms clumps within the nucleus. In patients with *C9orf72* mutations, this binding hinders how the protein regulates alternative splicing, a mechanism where specific portions of mRNA are edited out or reshuffled (Conlon et al., 2016). Now, a collaboration between the Columbia team and researchers at the New York Genome Center – including Erin Conlon as first author and James Manley as corresponding author – reports a new and unexpected twist to the story (Conlon et al., 2018).

The team hoped to use the splicing defects they identified in the mRNA targets of hnRNP H to create a ‘signature’ that would help them identify which cases are caused by mutations in *C9orf72*. To begin, they studied 18 of these targets in 50 postmortem samples from the brains of people with ALS, FTD, or both. None of the patients harbored *C9orf72* mutations, or any other known mutation. Unexpectedly, the mRNA sequences were still dysregulated in more than half of the samples: if there was no repeat RNA to trap hnRNP H, how could this be the case?

To answer this question, Conlon explored the solubility of hnRNP H in an effort to determine if it was sequestered or somewhat dysfunctional. The samples were split into two groups depending on whether they showed a splicing signature similar to the one present in *C9orf72* mutation cases. The team found that hnRNP H was twice as insoluble in the group with the splicing signature compared to the samples without these RNA splicing defects. What drives this insolubility when the mutations in *C9orf72* are absent?

Conlon et al. reasoned that hnRNP H could be corrupted by proteins with similar properties that had turned insoluble; and indeed, they discovered three other RNA-binding proteins which had become less soluble. They then turned their attention to one of these proteins, TDP-43, whose loss had been studied before (Polymenidou et al., 2011; Tollervey et al., 2011). This protein was distributed similarly in the brains of all the patients: in other words, histopathology (or looking at the samples under the microscope) was unable to distinguish between the two groups. However, when measuring the mRNA targets of TDP-43, it appeared that TDP-43 had lost much more of its function in the samples with the splicing signature that matches the *C9orf72* mutation carriers. The insolubility of TDP-43 (how it behaves biochemically) was therefore more important to identify the two groups.

Collectively, these findings support the idea that the splicing defects found in ALS and FTD appear when any of several RNA-binding proteins becomes insoluble. As such, Conlon et al. infer that we should reconsider how we view these conditions, and move away from the idea that they are simply TDP-43 diseases. Their model predicts the existence of an equilibrium between an array of RNA-binding proteins, each at a different concentration and in a physically distinct state. Even subtle mutations could change the concentration and the solubility of any of these proteins, which could tip the scale and culminate in various defects in RNA processing. Therapeutic interventions that reestablish the RNA-binding protein balance could significantly slow or stop the course of the diseases.

**Figure 1.**
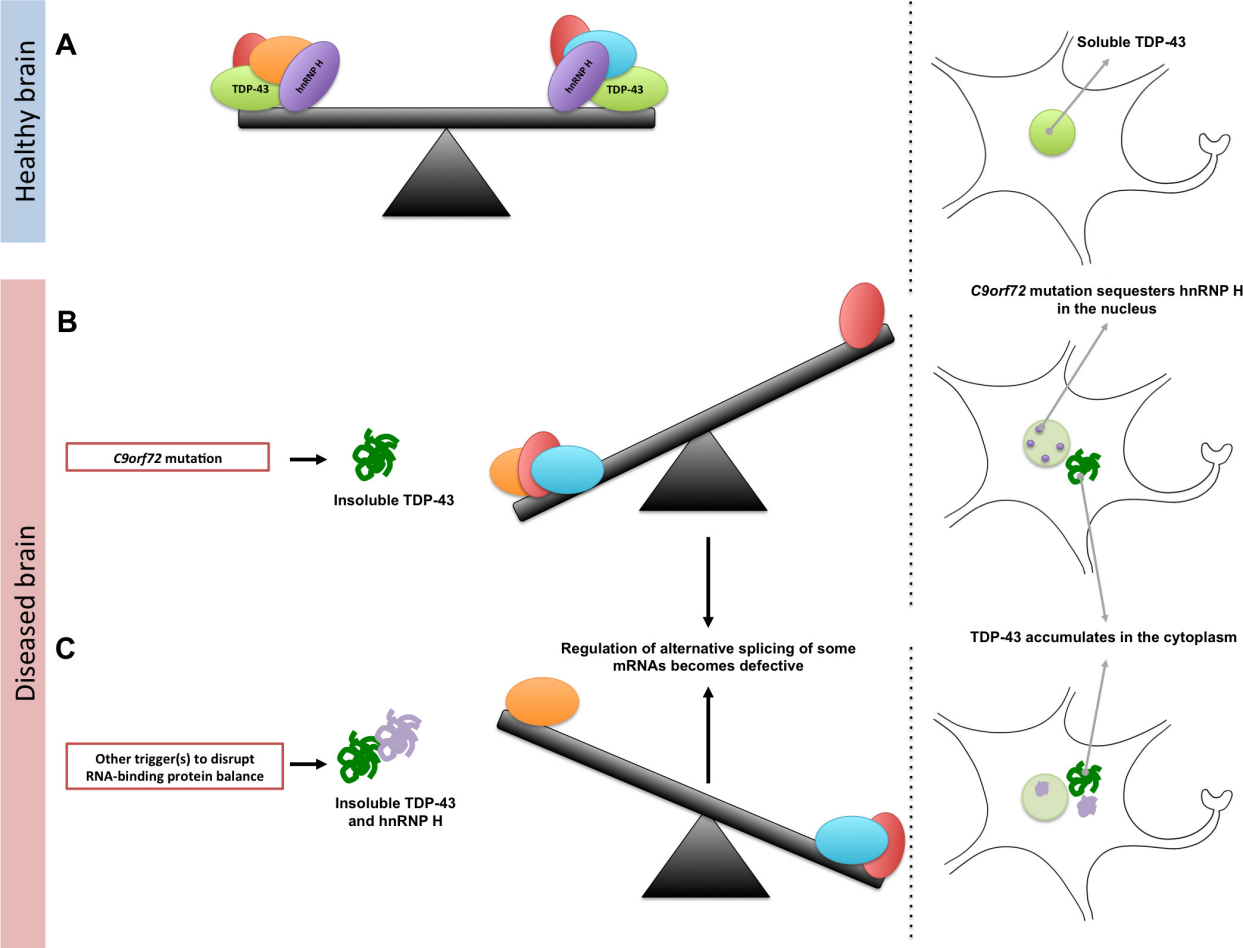
Maintaining the balance of RNA-binding proteins. (**A**) RNA-binding proteins (red, green, orange, purple, and blue), such as TDP-43 (green), regulate various RNA processing pathways, like the alternative splicing of mRNA. Many of these proteins are also prone to aggregating and becoming insoluble. As represented by the seesaw, this means that their concentration, solubility, and interactions with proteins must be controlled to maintain balance in the cell (right). Under normal conditions, TDP-43 is soluble and is mainly localized in the nucleus of the cell. (**B**) A mutation in a gene called *C9orf72* can result in a reduction in the solubility of an RNA-binding protein known as hnRNP H (purple). This can also lead to changes in the concentration and solubility of other RNA-binding proteins (including TDP-43), which disrupts alternative splicing and other processes in the cell. The reduction in the solubility of TDP-43 causes it to accumulate in the cytoplasm, which is a hallmark of ALS and FTD. (**C**) Other triggers, such as genetic and environmental factors, can change the concentration and solubility of RNA-binding proteins (unbalanced seesaw); this causes them to misfold, become insoluble and accumulate in the cytoplasm.
